# The role of the Sunda shelf biogeographic barrier in the cryptic differentiation of *Conus litteratus* (Gastropoda: Conidae) across the Indo-Pacific region

**DOI:** 10.7717/peerj.15534

**Published:** 2023-07-14

**Authors:** Shijin Ameri, Laxmilatha Pappurajam, K. A. Labeeb, Ranjith Lakshmanan, Kathirvelpandian P. V. Ayyathurai

**Affiliations:** 1 Central Marine Fisheries Research Institute, Ernakulam, Kerala, India; 2 School of Biosciences, Mangalore University, Mangalore, India; 3 Tuticorin Regional Station, CMFRI, Tuticorin, India; 4 PMFGR Centre, ICAR-NBFGR, Ernakulam, India

**Keywords:** Mitochondrial markers, Cryptic species, Ocean currents, Sunda shelf barrier, Benthic ecology, Conidae

## Abstract

Geographical and oceanographic processes have influenced the speciation of marine organisms. Cone snails are marine mollusks that show high levels of endemism and a wide distributional range across the Indian and Pacific Oceans. Discontinuities in distributions caused by biogeographic barriers can affect genetic connectivity. Here we analysed the connectivity within *Conus litteratus* using samples from the Lakshadweep archipelago (Arabian Sea, Indian Ocean) and from the Pacific Ocean. Maximum likelihood analyses based on the mitochondrial cytochrome *C* oxidase subunit I (COI) and on the non-coding 16S ribosomal RNA (16S rRNA) genes revealed cryptic diversity within *C. literatus* occupying distinct oceanographic regions. The intraspecific genetic distances between the two distinct clades of *C. literatus* from the Arabian Sea and the Pacific Ocean ranged from 7.4% to 7.6% for COI and from 2.4% to 2.8% for 16S rRNA genes, which is larger than the threshold limit for interspecific differentiation. The haplotype network analysis also corroborated the existence of two different lineages within *C. litteratus*. The detected genetic discontinuities reflect the effect of the Sunda shelf biogeographic barrier on the allopatric divergence of *C. litteratus*.

## Introduction

The Arabian Sea in the Indian Ocean harbors a wide range of marine ecosystems and its endemic biodiversity is strongly linked to many unique ecological and biogeographical features (Laxmilatha et al., 2021). Studies on biogeographical factors reveal the discontinuities in species distribution and genetic diversity of species. Major barriers which restrict larval dispersion and gene flow between oceanic macro ecosystems have previously been identified (Ekman, 1953; Briggs & Bowen, 2012). The mode of larval development is the road map of evolutionary studies in the marine realm and plays an important role in connectivity (Palumbi, 1994; Servedio & Noor, 2003; Beger et al., 2014; Tiego et al., 2017).

The west coast of India is one of the most productive seas in the Indian Ocean, with strong seasonal upwelling resulting in high levels of primary productivity (Kumar et al., 2000) that shapes unique marine biodiversity in the eastern Arabian Sea, including the Lakshadweep archipelago (Ameri et al., 2022). The genetic structure of marine benthic invertebrate populations and their larval dispersal are known to be greatly influenced by oceanographic and geographical processes (Palumbi, 1994; Cunha et al., 2005; Whittaker & Fernández-Palacios, 2007). Several mitochondrial-based studies have revealed the role that biogeography and barriers have played in the dispersal and divergence of coral reef fish lineages (Fessler & Westneat, 2007). Recent phylogeographic studies revealed the existence of cryptic differentiation within South Pacific coral reef fish species due to the influence of soft biogeographic barriers (Drew & Barber, 2012). Due to a biogeographic break located at 30°S in the southeast Pacific, several marine benthic invertebrate taxa exhibit strong genetic differentiation in this area. This indicates that biogeographic barriers often limit gene flow, promoting divergence and speciation (Haye et al., 2014, 2019). Genetic diversity in Chilean barnacles also reflects the role of a biogeographic transition zone (Zakas et al., 2009). The Sunda shelf barrier (SSB), is a shallow continental shelf among Indonesian islands that restricts exchange between the tropical Indian Ocean and the western Pacific during low sea level stands (Ekman, 1953; Randall, 1998). Significant genetic structure due to the influence of the SSB has been detected in pelagic coral reef fishes (Bay et al., 2004; Bowen et al., 2016).

Cone snails have separate sexes, and produce eggs that typically adhere to a hard surface and undergo internal fertilization (Kohn, 1959). The type of larval development has a significant impact on the dispersal abilities of these venomous marine snails (Cunha et al., 2005). Larvae exhibiting nonplanktonic lecithotrophic developmental mode have no pelagic phase, while planktotrophic larvae may remain in the water column for large periods (Jablonski, 1986). The present study aims to analyze the genetic diversity of *Conus litteratus* Linnaeus, 1758 (Gastropoda, Conidae) from the Lakshadweep archipelagos in the Indian Ocean.

## Materials and Methods

### Sample collection and morphological analyses

**Figure 1:**
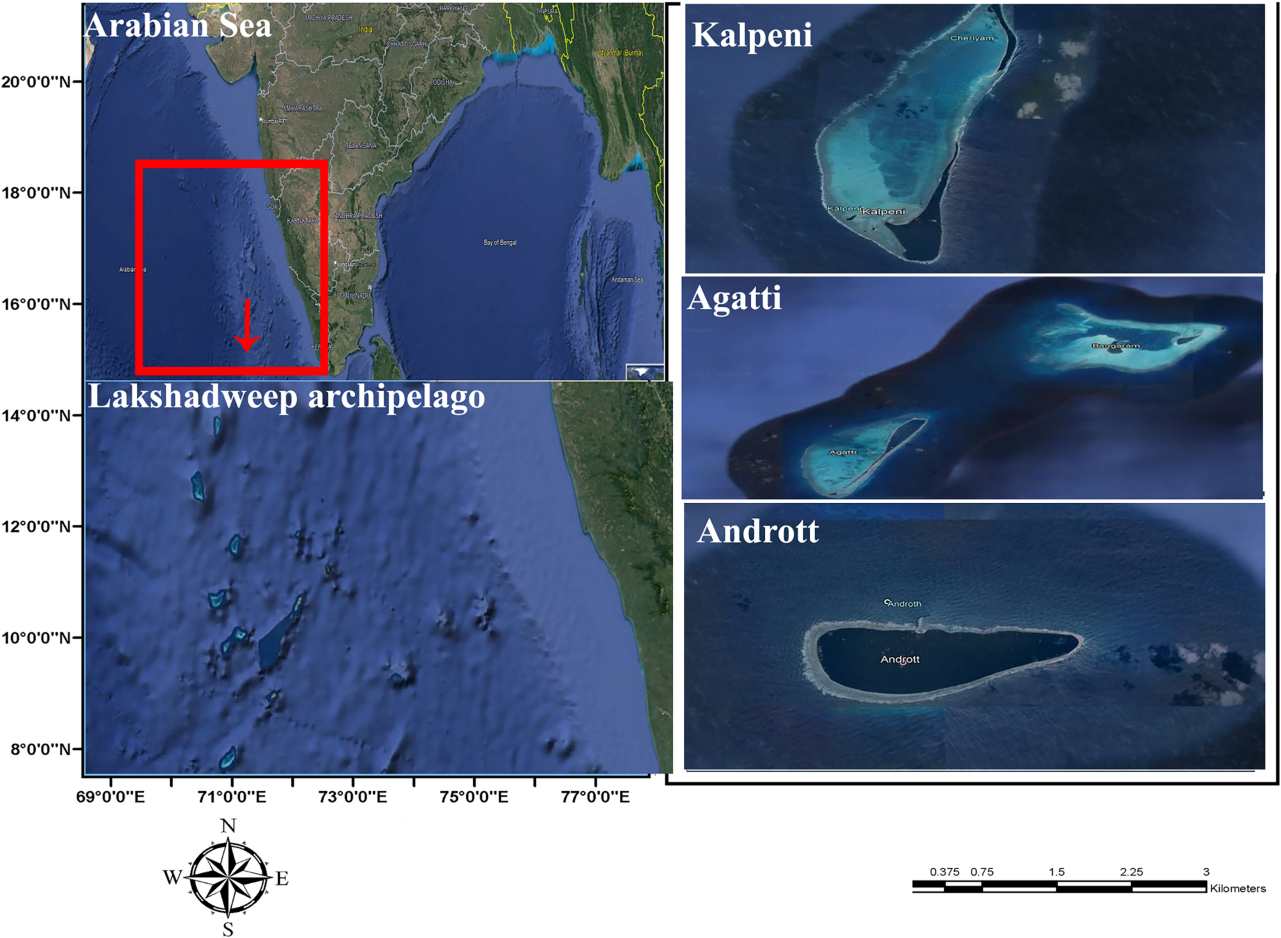
Sample collection location, Islands of Lakshadweep archipelago, Arabian Sea, Indian Ocean.

Five specimens were collected from the lagoon of Agatti, Andrott and Kalpeni Islands of Lakshadweep archipelago, Arabian Sea (Fig. 1) by snorkeling after obtaining field permit from the Lakshadweep administration. Tissue samples from the foot muscle were preserved in 95% ethanol. Taxonomy based on the following morphological characters of the shell (color, standard length (SL); shell width (SW); aperture height (AH); relative spire height (RSH = (SL − AH)/SL); position of the maximum diameter (PMD) = HMD/AH) followed Röckel, Korn & Kohn (1995).

### DNA extraction and PCR amplification

DNA was isolated from the preserved tissue using a customized marine premium kit Origin ORIonX (ODP324 02P), according to the manufacturer’s instructions. The purity and quantity of isolated DNA were tested by Nanodrop (Eppendorf Pvt. Ltd, Chennai, Tamilnadu, India). The partial portion of the mitochondrial gene cytochrome *c* oxidase subunit I (COI) gene was amplified using the universal primers LCO1490/HCO2198 (Folmer et al., 1994). A fragment of the ribosomal 16S rRNA was amplified using the 16Sar/16Sbr primers (Palumbi, 1996). All PCR reactions were performed as previously described in Laxmilatha et al. (2021) and Ameri et al. (2022) with slight modifications. Reaction volume of 25 μl, containing 40 ng of DNA, 2.5 mM MgCl_2_ and 0.3 mM of each primer. The amplification reaction consisted of an initial denaturation step at 94 °C for 5 min, followed by 35 cycles of denaturation at 95 °C for 30 s, annealing for 35 s at 48 °C for COI and 52 °C for 16S rRNA with an extension at 72 °C for 1 min. The final extension was at 72 °C for 5 min.

### Phylogenetic analyses

COI and 16S rRNA sequences of the five specimens from the Arabian Sea were submitted to GenBank, NCBI and accession numbers were obtained. COI and 16S rRNA sequences from the Pacific specimens were retrieved from NCBI database (Table 1). All the sequences (600 bp for COI and 477 bp for 16S rRNA) were aligned in a final data set using the software BIO Edit (Hall, 1999). A maximum likelihood (ML) analysis (1,000 bootstrap) with Kimura-2-parameters (K2P) was performed for each gene, separately using MEGA 7 (Kumar, Stecher & Tamura, 2016). Based on previous studies (Puillandre et al., 2014), *Californiconus californicus* was selected as the outgroup.

**Table 1:** *C. litteratus* Specimens used for phylogenetic analyses.

Sl. No	Species	Specimen voucher	Location	NCBI accession numbers	
				COI	16S rRNA
1	*C. litteratus*	LD008.1	Agatti, Lakshadweep Arabian Sea, Indian Ocean	ON942252	ON943042
2	*C. litteratus*	LD008.2	Agatti, Lakshadweep Arabian Sea, Indian Ocean	ON942253	ON943043
3	*C. litteratus*	LD008.3	Kalpeni, Lakshadweep, Arabian Sea, Indian Ocean	ON942254	ON943044
4	*C. litteratus*	LD008.4	Kalpeni, Lakshadweep, Arabian Sea, Indian Ocean	ON942255	ON943045
5	*C. litteratus*	LD008.5	Kavaratti, Lakshadweep, Arabian Sea, Indian Ocean	ON942256	ON943046
6	*C. litteratus*	_	Pacific Ocean	KJ550336	EU794329
7	*C. litteratus*	MNHN:IM-2007-30755	Pacific Ocean	KJ550338	KJ550867
8	*C. litteratus*	_	Pacific Ocean	KJ550337	HM212495
9	*C. litteratus*	UF303218	Pacific Ocean	KJ549941	KJ550652

A timetree was inferred using the Realtime method ML (Tamura et al., 2012) and the Hasegawa-Kishino-Yano model HKY G+I. The best-fit model was selected using IQ tree model finder (Kalyaanamoorthy et al., 2017). The timetree was computed using calibration constraints obtained from previous studies (Williams & Duda, 2008) and also confirmed with Timetree database (http://www.timetree.org) (Kumar et al., 2017). The estimated log-likelihood value is −1,671.9546. A discrete Gamma distribution was used to model evolutionary rate differences (+*G*, parameter = 200.0000).

### Population genetic structure

Haplotype and nucleotide diversity (π) based on COI and 16S rRNA sequences were calculated using the program PopART (Leigh & Bryant, 2015). A median-joining haplotype network (Bandelt, Forster & Röhl, 1999) was constructed to visualize the relationships among haplotypes, using the program PopART (Leigh & Bryant, 2015) with default settings. An analysis of molecular variance (AMOVA) (Excoffier, Smouse & Quattro, 1992) was used to test for hierarchical population genetic structure among regions using 1,000 permutations using the program PopART (Leigh & Bryant, 2015).

## Results

### Morphological analyses

**Figure 2:**
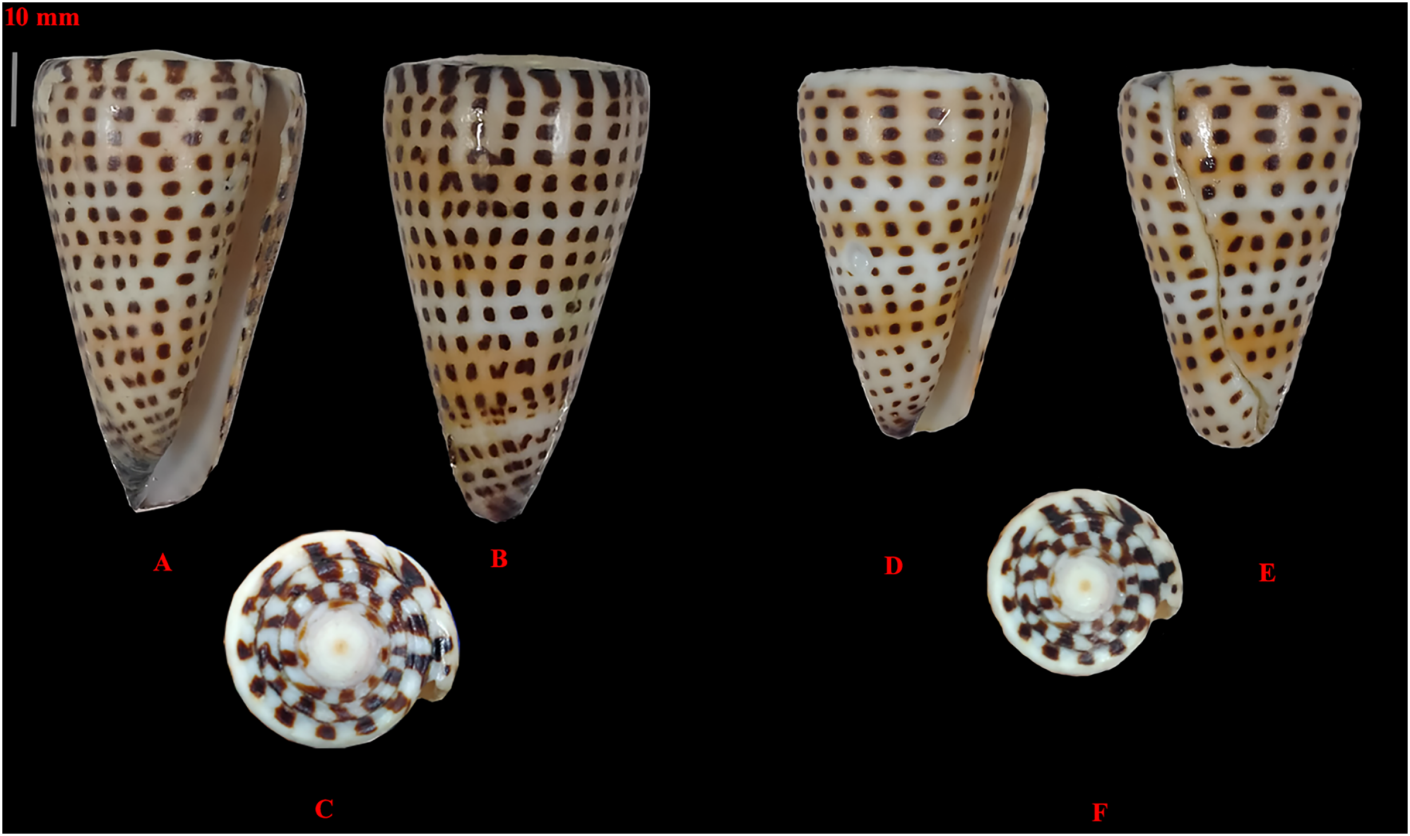
Images of two specimens of *Conus litteratus* collected from the Lakshadweep archipelago. (A–C) Specimen 1, haplotype 1 and (D–F) specimen 2, haplotype 2.

The morphological variation within the collected *C. litteratus* samples (Fig. 2) was the following: standard length from 51 to 59 mm; shell width from 31 to 32 mm; aperture height from 50 to 58 mm; relative diameter from 0.53 to 0.62; relative spire height of 0.01; position of the maximum diameter from 0.82 to 0.86 mm.

### Phylogenetic analyses

**Figure 3:**
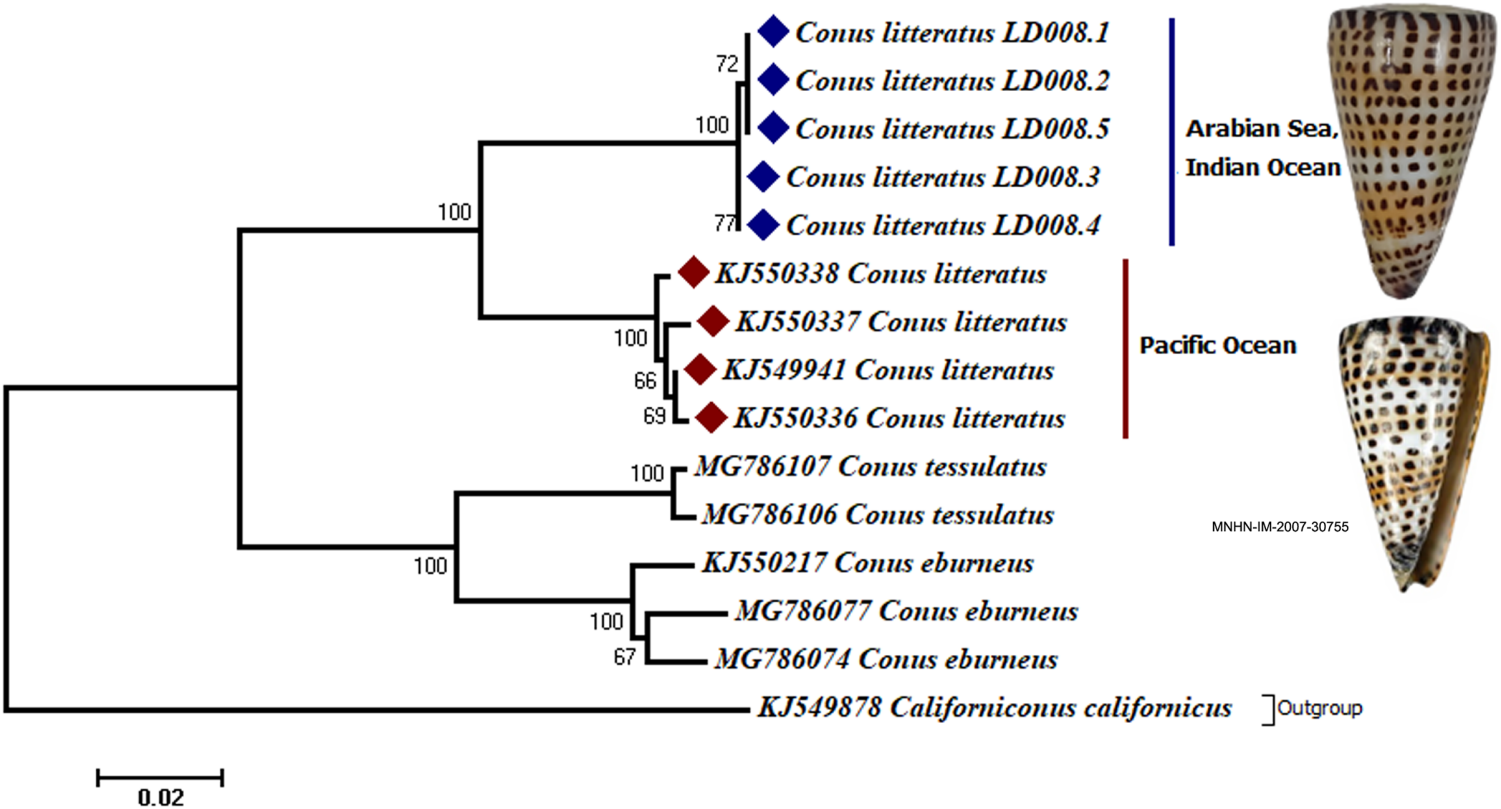
Phylogenetic tree of *C. litteratus* from the Arabian Sea and the Pacifc Ocean based on the cytochrome oxidase subunit I gene. The image of *C. litteratus* from the Pacific Ocean was retrieved from the Muséum National d’Histoire Naturelle, Paris.

**Figure 4:**
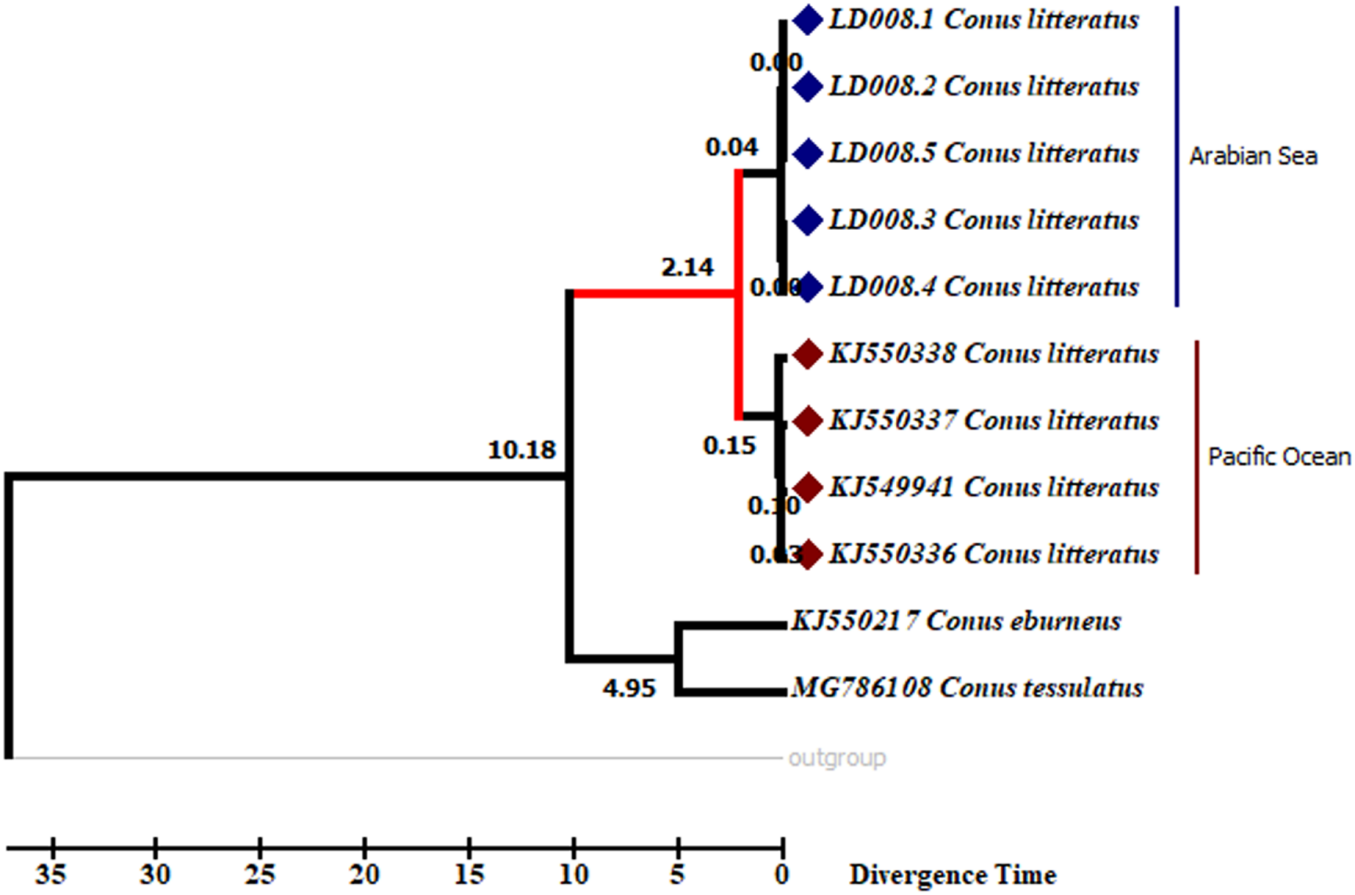
Divergent timetree of the Pacific and Arabian Sea specimens of *C. litteratus*. The number of nodes indicates the time of the divergent of the clusters in million years ago (MYA).

Analyses of the partial COI sequences revealed two well-differentiated clades within *C. litteratus*, one including specimens of the Arabian Sea and the other with samples from the Pacific Ocean (Fig. 3). The partial 16S rRNA gene sequences yielded an identical phylogenetic tree (BS 98) (Fig. S1). The dating analysis reveals that *C. litteratus* of Arabian Sea specimens diverged at 2.14 MYA (Fig. 4).

### Population genetic analyses

**Figure 5:**
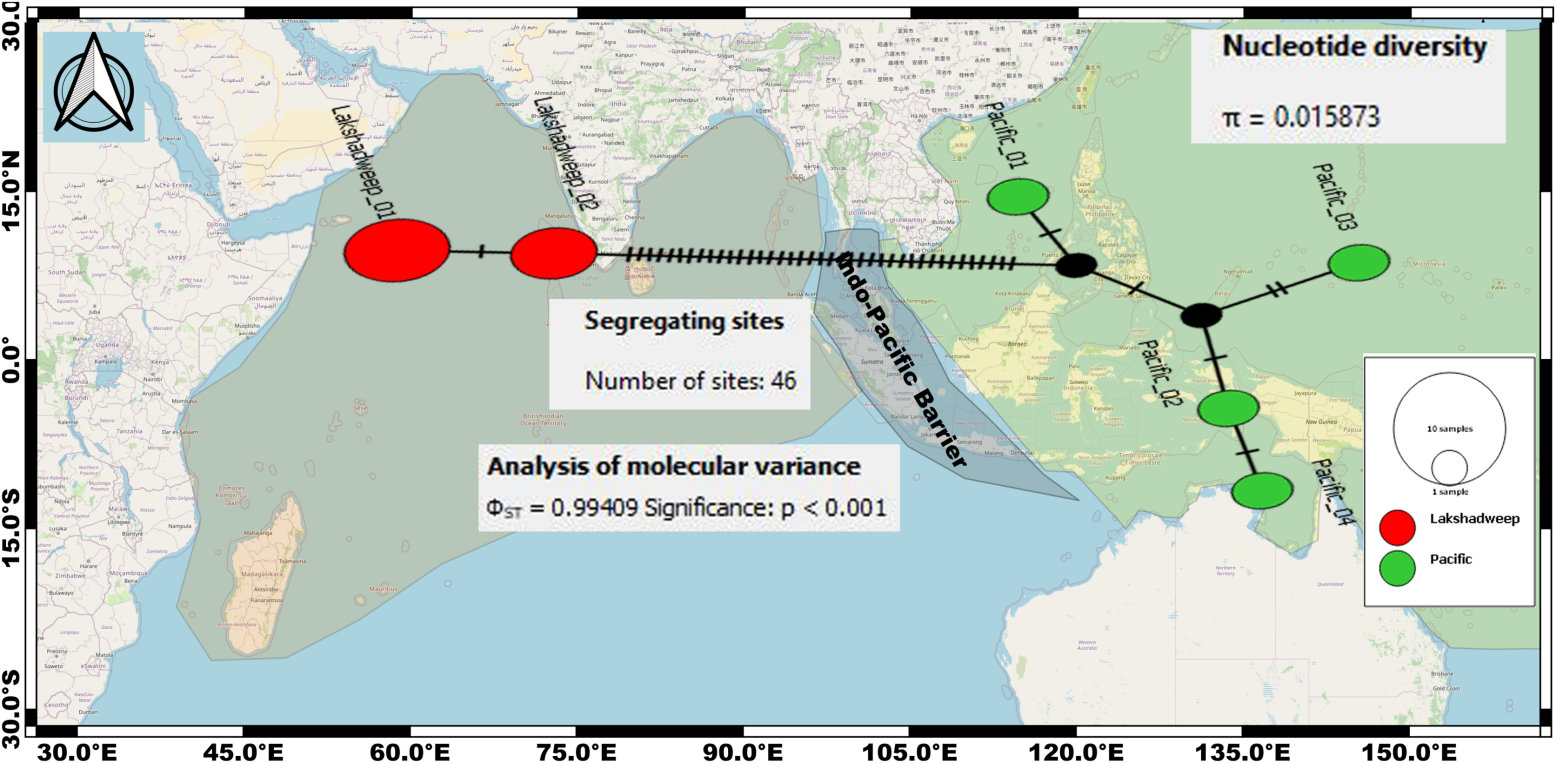
Median-joining networks for representing the genetic divergence (cryptic species) among the *C. litteratus* specimens of Indian Ocean (Red dots) and Pacific Ocean (Green dots) based on COI sequences. Circle sizes correspond to haplotype frequencies. Dashed line represents one mutational step between the haplotypes (39 segregating sites differentiating Lakshadweep and Pacific populations).

The intraspecific K2P genetic distance between the Arabian Sea and the Pacific Ocean *C. litteratus* specimens ranged from 7.4% to 7.6% and from 2.4% to 2.8% based on COI (Table 2) and 16S rRNA (Table 3), respectively. The AMOVA based on COI sequences shows high genetic diversity within *C. litteratus* (ϕ_ST_ = 0.99409; *P* < 0.001) and revealed 46 segregating and 39 segregating sites differentiating Lakshadweep and Pacific populations, respectively (Fig. 5). The AMOVA based on 16S rRNA sequences (ϕ_ST_ 0.98703; *P* < 0.001), shows 13 and 11 segregating sites differentiating Lakshadweep and Pacific populations, respectively (Fig. S2).

**Table 2:** Kimura 2 parameter (K2P) genetic distances based on COI.

*Conus_litteratus_LD008.1*													
*Conus_litteratus_LD008.2*	0.000												
*Conus_litteratus_LD008.3*	0.002	0.002											
*Conus_litteratus_LD008.4*	0.002	0.002	0.000										
*Conus_litteratus_LD008.5*	0.000	0.000	0.002	0.002									
*KJ550338_Conus_litteratus*	0.074	0.074	0.072	0.072	0.074								
*KJ549941_Conus_litteratus*	0.076	0.076	0.074	0.074	0.076	0.005							
*KJ550337_Conus_litteratus*	0.078	0.078	0.076	0.076	0.078	0.007	0.005						
*KJ550336_Conus_litteratus*	0.078	0.078	0.076	0.076	0.078	0.007	0.002	0.007					
*MG786077_Conus_eburneus*	0.151	0.151	0.149	0.149	0.151	0.158	0.160	0.167	0.158				
*MG786074_Conus_eburneus*	0.151	0.151	0.149	0.149	0.151	0.158	0.156	0.162	0.158	0.022			
*KJ550217_Conus_eburneus*	0.151	0.151	0.149	0.149	0.151	0.151	0.149	0.156	0.151	0.028	0.019		
*MG786107_Conus_tessulatus*	0.149	0.149	0.151	0.151	0.149	0.147	0.145	0.147	0.147	0.087	0.077	0.075	
*MG786106_Conus_tessulatus*	0.151	0.151	0.153	0.153	0.151	0.149	0.147	0.149	0.149	0.085	0.075	0.073	0.005

**Table 3:** Kimura 2 parameter (K2P) genetic distances based on 16s rRNA.

*Conus_litteratus_LD008.1*												
*Conus_litteratus_LD008.2*	0.000											
*Conus_litteratus_LD008.3*	0.000	0.000										
*Conus_litteratus_LD008.4*	0.000	0.000	0.000									
*Conus_litteratus_LD008.5*	0.000	0.000	0.000	0.000								
*EU794329_Conus_litteratus*	0.024	0.024	0.024	0.024	0.024							
*KJ550867_Conus_litteratus*	0.028	0.028	0.028	0.028	0.028	0.004						
*HM212495_Conus_litteratus*	0.024	0.024	0.024	0.024	0.024	0.000	0.004					
*KJ550652_Conus_litteratus*	0.024	0.024	0.024	0.024	0.024	0.000	0.004	0.000				
*KJ550825_Conus_eburneus*	0.068	0.068	0.068	0.068	0.068	0.061	0.061	0.061	0.061			
*KJ550607_Conus_eburneus*	0.071	0.071	0.071	0.071	0.071	0.064	0.064	0.064	0.064	0.002		
*KJ550608_Conus_eburneus*	0.068	0.068	0.068	0.068	0.068	0.061	0.061	0.061	0.061	0.004	0.006	
*KJ550735_Conus_tessulatus*	0.078	0.078	0.078	0.078	0.078	0.068	0.071	0.068	0.068	0.026	0.024	0.028

## Discussion

An especially interesting land barrier, the Sunda Shelf Barrier (SSB) between the Indian and Pacific Oceans shapes unique marine biodiversity in tropical Indian and Pacific Ocean regions (Randall, 1998). This Indo-Pacific barrier formed during glacial periods when the sea level drops expose a complete land wall between the Indian and western Pacific Oceans (Ekman, 1953; Randall, 1998). Recent phylogeographic studies have revealed cryptic diversity within South Pacific coral reef fishes promoted by soft biogeographic barriers (Fessler & Westneat, 2007; Drew & Barber, 2012). Bullethead parrotfish and seahorses from the Indo-Pacific region show significant genetic differences among locations separated by the Indo-Pacific SSB (Bay et al., 2004; Lourie & Vincent, 2004). The present study provides new insights into the role of the SSB in the divergence of a gastropod species, *C. litteratus*. Our analysis showed two distinct lineages of *C. literatus*, one from the Pacific and the other in the Arabian Sea. The two lineages did not exhibit any significant differences at the morphological level. The intraspecific genetic distance of *C. litteratus* between the Arabian Sea and Pacific Ocean lineages is larger than the threshold limit for different Conidae species (>6% in COI and >2% in 16S rRNA) (Puillandre et al., 2014). There is an increasing number of molecular-based studies showing the existence of cryptic diversity, in the absence of diagnostic morphological characters, particularly in gastropods (Duda et al., 2008; Puillandre et al., 2014).

The dispersal range and rate of speciation of cone snails may be significantly influenced by the different developmental modes that may range from planktotrophy (pelagic larval stage) to lecithotrophy (absence of pelagic larval stage), particularly in the archipelagic ecosystem (Kohn & Perron, 1994). For instance, in the Cape Verde archipelago all endemic species of *Conus* exhibit a nonplanktonic lecithotrophic developmental mode (Kohn & Perron, 1994; Cunha et al., 2005), which limits gene flow between populations because of the reduced capacity of the larvae to disperse. It is not possible from our data to determine whether the ancestors of *C. litteratus* of the Lakshadweep archipelago had planktotrophic larval development or non-planktonic lecithotrophy. However, insights from previous studies in cone snails confirm that most isolated oceanic islands show high levels of endemism promoted by nonplanktonic lecithotrophy (Kohn & Perron, 1994; Cunha et al., 2005, 2008).

Our dating analysis revealed that the two lineages of *C. litteratus* diverged during the Pleistocene at 2.14 MYA (Fig. 5). Earlier research indicated that the isolation of marginal ocean basins during the Pleistocene low sea-level stands may have promoted speciation within the Indo-West Pacific islands (McManus, 1985; Potts, 1985). The exchange of water between the west Pacific and Indian Oceans was averted during the Pleistocene due to the formation of a biogeographic barrier, the SSB, when the land area was maximal (Voris, 2000; Bird, Taylor & Hunt, 2005). The role of other biogeographic barriers in lineages’ divergence has been described in the Isthmus of Panama where the exchange of water between the tropical Atlantic and Pacific Oceans ceased about 3 million years ago promoting diversification of the existing fauna (Leigh, O’Dea & Vermeij, 2013). The broad distribution of *C. litteratus* throughout the Indian and Pacific Oceans makes this species an excellent model organism for studying patterns of species diversification. Our phylogenetic and phylogeographic analyses detected high levels of genetic differentiation between the two-ocean basis most likely due to the influence of SSB on *C. litteratus* dispersal during Pleistocene low sea level stands.

## Conclusion

The present study provides evidence for the existence of cryptic diversification within *C. litteratus* from the Lakshadweep archipelago. Molecular sequence analyses revealed the existence of two well-differentiated clades in specimens of *C. litteratus* from the Indian and Pacific Oceans. The intraspecific genetic distance between specimens of *C. litteratus* from the two ocean basins is larger than the threshold limit for interspecific differentiation, nonetheless further analyses based on nuclear markers are needed to confirm the taxonomic status of the species. Dating analyses revealed that the divergence of the two lineages occurred during the Pleistocene and was promoted by the SSB oceanographic barrier, when the land area was maximal.

## Supplemental Information

10.7717/peerj.15534/supp-1 Supplemental Information 1 Raw data of 16S rRNA sequences used for the study.

10.7717/peerj.15534/supp-2 Supplemental Information 2 Raw data of COI sequences used for the study.

10.7717/peerj.15534/supp-3 Supplemental Information 3 Morphological parameters of *C. litteratus* specimens.

10.7717/peerj.15534/supp-4 Supplemental Information 4 Phylogenetic tree based on 16srRNA gene representing a genetic variant of *C. litteratus* of the Arabian Sea and the Pacific Ocean.

10.7717/peerj.15534/supp-5 Supplemental Information 5 Median-joining networks for representing the genetic divergence (cryptic species) among the *C.litteratus* specimens of Indian Ocean (Red dots) and Pacific Ocean (Green dots) based on 16S rRNA. Circle sizes correspond to haplotype frequencies. Dashed line represents one mutational step between the haplotypes (11 segregating sites differentiating Lakshadweep and Pacific populations).

## Additional Information and Declarations

### Field Study Permissions

The following information was supplied relating to field study approvals (*i.e*., approving body and any reference numbers): Field permits were obtained from the Lakshadweep administration.

### DNA Deposition

The following information was supplied regarding the deposition of DNA sequences: The sequences are available in the Supplemental Files and at GenBank: COI (ON942252 to ON942256), 16SrRNA (ON943042 to ON943046).

### Data Availability

The following information was supplied regarding data availability: The raw data used for the morphological analysis are available in the Supplemental Files.
