# Supplementary figures and images for: The role of the Sunda shelf biogeographic barrier in the cryptic differentiation of Conus litteratus (Gastropoda: Conidae) across the Indo-Pacific region
Source: PeerJ. 2023 Jul 14;11:e15534. doi: 10.7717/peerj.15534 (PMC10351507; doi:10.7717/peerj.15534)

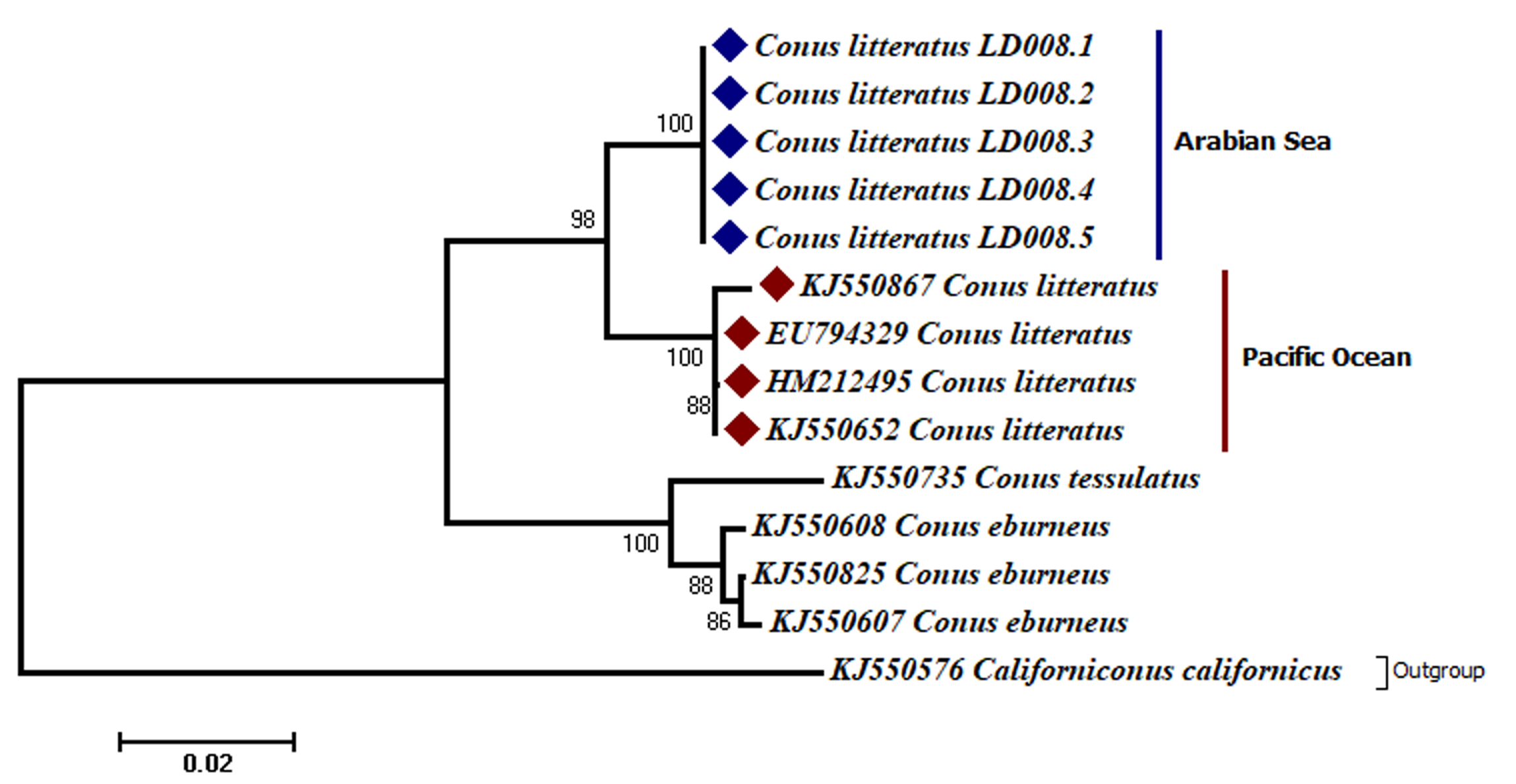

Supplement: Supplemental Information 4 [file peerj-11-15534-s004.png]

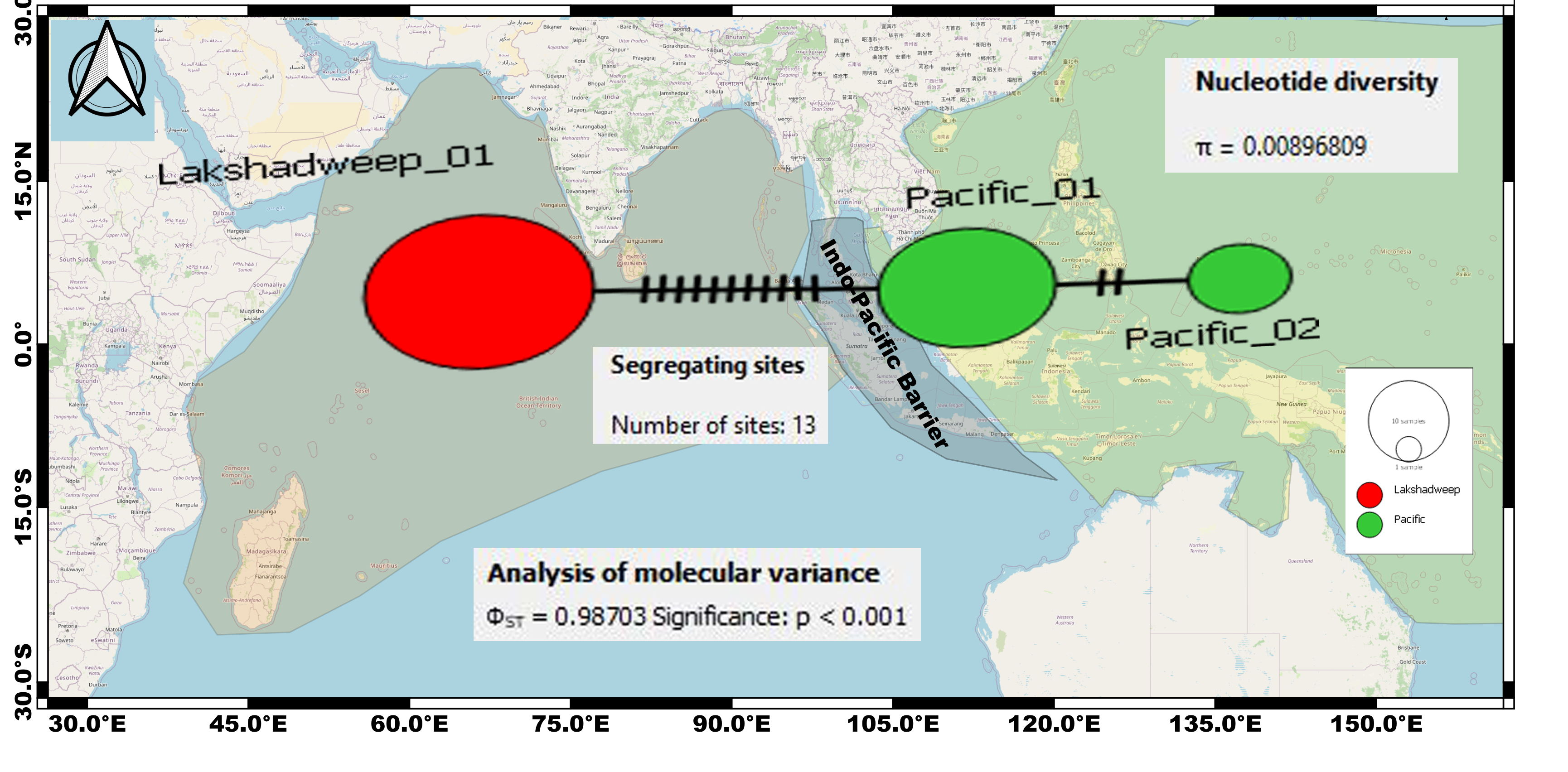

Supplement: Supplemental Information 5 — Circle sizes correspond to haplotype frequencies. Dashed line represents one mutational step between the haplotypes (11 segregating sites differentiating Lakshadweep and Pacific populations). [file peerj-11-15534-s005.png]
